# The association of healthy lifestyle index score and the risk of renal cell cancer in the Netherlands cohort study

**DOI:** 10.1186/s12885-023-10627-6

**Published:** 2023-02-16

**Authors:** Romain Meer, Jeroen van de Pol, Piet A. van den Brandt, Leo J. Schouten

**Affiliations:** 1 Department of Epidemiology, GROW- School for Oncology and Reproduction, Maastricht University, Peter Debyeplein 1, 6229 HA Maastricht, The Netherlands; 2 Department of Epidemiology, Care and Public Health Research Institute (CAPHRI), Maastricht University, Maastricht, The Netherlands

**Keywords:** Renal cell cancer, Healthy lifestyle, Prospective cohort study, Risk factors

## Abstract

**Background:**

Diet, alcohol, cigarette smoking, physical inactivity, and body mass index have been studied as risk factors for renal cell cancer (RCC). The joint effects of these lifestyle factors, captured as Healthy Lifestyle Index (HLI), were examined in one previous study. This study aims to investigate the association between HLI score and RCC risk in the prospective Netherlands Cohort Study (NLCS).

**Methods:**

A case-cohort analysis (3,767 subcohort members, 485 cases) was conducted using NLCS data (*n* = 120,852). Data on aforementioned risk factors was used to calculate HLI score, ranging 0–20, with higher scores reflecting healthier lifestyles. RCC occurrence was obtained by record linkage to cancer registries. Multivariable-adjusted proportional hazard models were used to calculate Hazard Ratios (HR) and 95% Confidence Intervals (95%CI).

**Results:**

Compared to participants in the unhealthiest HLI category, participants within the healthiest category had a lower RCC risk (HR = 0.79, 95%CI = 0.56–1.10, *p* for trend 0.045). A standard deviation (± 3-unit) increase in HLI score was not statistically significantly associated with a lower RCC risk (HR = 0.92, 95%CI = 0.83–1.01). This association was stronger after excluding diet or alcohol from the score, although confidence intervals overlap.

**Conclusions:**

Adherence to a healthy lifestyle was weakly, though not statistically significantly, associated with a lower RCC risk.

**Supplementary Information:**

The online version contains supplementary material available at 10.1186/s12885-023-10627-6.

## Background

Renal cell cancer (RCC) is the most common form of kidney cancer with an estimated global age-standardized incidence rate of 4.4 cases per 100,000 individuals in 2018 [1–3]. Over the last decades, various RCC risk factors have been identified through case–control studies and cohort studies. RCC risk factors related to demography are male sex and age [2, 4–6], whereas risk factors related to medical history are hypertension, diabetes, kidney stones and chronic kidney disease [2, 6, 7]. Lastly, several risk factors related to lifestyle have been studied for RCC. Cigarette smoking and a high body mass index (BMI) were associated with an increased RCC risk [2, 7–11]. Unhealthy diet and physical inactivity have been identified as a risk factor in various cancers [2, 12], but its roles in RCC remain speculative [2, 13–15]. Finally, multiple meta-analyses have summarized evidence on alcohol consumption as RCC risk factor. Moderate alcohol consumption was associated with an increased risk of several forms of cancer, but an inverse association was found in RCC [7, 16–18].

Several studies have used a Healthy Lifestyle Index (HLI), consisting of diet, cigarette smoking, alcohol consumption, physical activity and BMI, to examine the association between a healthy lifestyle and the risk of various types of cancers [19–23]. The chosen components are modifiable risk factors, that are associated with the risk of cancer and other lifestyle-related chronic diseases (i.e. diabetes mellitus, hypertension and coronary heart diseases) [24, 25].

To illustrate, McKenzie and colleagues studied the association between HLI score and the risk of subgroups of cancer, such as tobacco-related cancers [19]. The researchers observed that higher HLI scores, which reflect healthier lifestyles, were associated with lower risks of cancer subgroups. Evidence from other studies have demonstrated that risks of ovarian cancer, endometrial cancer, pancreatic cancer and breast cancer were lower in individuals with high HLI scores [20–22]. Nevertheless, to the best of our knowledge, only one previous study has specifically examined the association between HLI score and RCC risk [23]. In that study, a Norwegian prospective cohort study, a higher Healthy Lifestyle Index score was associated with a lower RCC risk.

The first aim of this study was to examine the association between HLI score, adapted from the McKenzie study [19], and RCC risk. The second aim was to study individual contributions of lifestyle risk factors to RCC risk by in turn eliminating each lifestyle component from the HLI. The goal of this aim was to examine whether the association between HLI score and RCC risk was stronger when unestablished lifestyle factors, such as diet and physical activity, were omitted from the HLI. Finally, we also want to investigate whether hypertension is an intermediate factor of HLI score and RCC risk, since lifestyle is also associated with hypertension, and hypertension is a risk factor of kidney cancer. This study may shed light on the joint influence of lifestyle factors on RCC risk.

## Materials & methods

### Study population and design

This study used a case-cohort analysis using data from The Netherlands Cohort Study on Diet and Cancer (NLCS) (*n* = 120,852). The NLCS is a nation-wide prospective cohort study initiated in September 1986 with the inclusion of 58,279 men and 62,573 women between the ages of 55–69 years. Further details of the NLCS have been described in Additional file 1: Appendix 1.1 and elsewhere [26]. A subcohort of 5,000 participants was randomly sampled out of the full cohort at baseline to make follow-up and data processing more efficient. The full cohort was followed up for cancer occurrence via computerized record linkage with the Netherlands Pathology Registry (PALGA), the Netherlands Cancer Registry (NCR) and the cause of death registration maintained by Statistics Netherlands [27]. Members of the subcohort were followed up biennially until the year 2000 using mailed questionnaires for migration status and vital status. In case of no response, vital status and migration status were obtained by contacting municipal registration registries. After the year 2000, follow-up was conducted by record linkage to automated population registries. After 20.3 years, follow-up data of vital status of subcohort members was nearly 100% complete. Moreover, completeness of cancer status through record linkage was estimated to be approximately 96% [28]. Approval for the NLCS was granted by institutional review boards from Maastricht University in 1985, and the Netherlands Organization for Applied Scientific Research in 1986. Informed consent to participate was taken from all participants to participate in the study.

**Fig. 1:**
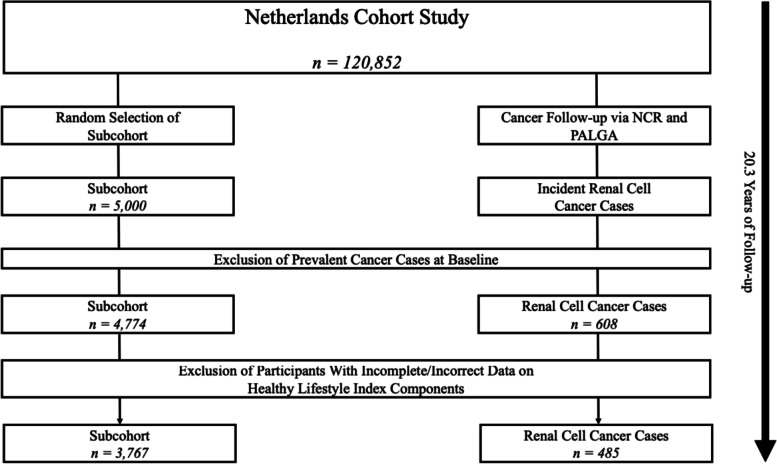
Flowchart of study participant selection and follow-up within the Netherlands Cohort Study (1986–2006). Footnote: NCR = Netherlands Cancer Registry, PALGA = Netherlands Pathology Registry

Between 1986 and 2006, 608 histologically verified RCC cases (International Classification of Diseases for Oncology 3 (ICD-O-3) C64.9) were identified. Cohort members were excluded from data analysis when they had prevalent cancer at baseline, with exception of skin cancer, had inconsistent information on exposure variables, had incomplete or inconsistent information in questions on dietary intake [29], or had missing data on exposure variables. As a result, the final study population for this study consisted of 3,767 subcohort members and 485 cases (Fig. 1).

### Exposure and covariate ascertainment

Data regarding exposures and covariates were collected from all cohort members using a self-administered questionnaire at baseline. The questionnaire included a 150-item semi-quantitative food frequency questionnaire (SFFQ) focusing on dietary habits of the participant within the last year. The validity and reliability of the SFFQ have been tested within the NLCS in a period of respectively two and five years after baseline and were both shown to be adequate [29, 30]. Average daily intake of nutrients was calculated from the SFFQ data using the 1986 Dutch food composition table [31]. Data obtained via the SFFQ was used to calculate HLI component scores for diet and alcohol consumption. The diet component score was based on energy-adjusted intake of six dietary components: fibre, red and processed meat, the ratio of poly-unsaturated to saturated fat, trans-fats, glycaemic load, and fruits and vegetables. Detailed information on the construction of the HLI diet component score is described in Additional file 1: Appendix 1.2. The non-SFFQ part of the questionnaire was used to collect data on the other three HLI components. The respondent was asked to provide information on non-occupational PA (based on frequency and duration of cycling, walking, gardening, sports), smoking habits (based on current smoking status, smoking frequency, and years of abstinence) and anthropometric data (height and weight at baseline) needed for the calculation of BMI.

Moreover, respondents were asked to provide information on potential confounders and effect-modifiers, like history of hypertension. The participant was labelled as hypertensive when the participant self-reported a physician’s diagnosis of hypertension and/or an antihypertensive drug administration of at least six months. Other potential confounders were level of completed education, daily intake of three salts (sodium, potassium, and magnesium; obtained via SFFQ), history of diabetes, history of kidney stones and familial history of (renal) cancer.

### Healthy lifestyle index

HLI scores were calculated using data from five components measured at baseline: diet, alcohol consumption, smoking habits, non-occupational PA, and BMI. Each component contributed 0 (unhealthiest habit) to 4 (healthiest habit) points to the HLI score. Similar to McKenzie et al. [19], five categories of approximately equal size were created for each component to reflect variation in lifestyle as much as possible. Sex-specific quintiles were created for diet and physical activity, while for BMI and smoking the same cutoff points were used as McKenzie et al. Because the NLCS participants hardly consumed > 60 g/day, we used the classification that is used in other NLCS publications. (e.g.: [32]) Detailed description of computation of HLI scores and cut-off values for each component are stated in Additional file 1: Appendix 1.2 and in Table A1, respectively.

**Table 1:** Baseline characteristics of the subcohort and renal cell cancer cases in the Netherlands Cohort Study on Diet and Cancer (1986–2006)

Baseline Characteristics	Subcohort (*n* = 3767)		RCC Cases (*n* = 485)	
HLI Total Category				
1 “Unhealthiest Lifestyle”	541	(14.4)	84	(17.3)
2 “Moderately Unhealthy Lifestyle”	1023	(27.1)	158	(32.6)
3 “Moderately Healthy Lifestyle”	1352	(35.9)	157	(32.4)
4 “Healthiest Lifestyle”	851	(22.6)	86	(17.7)
Demographic Characteristics				
Age at Baseline (years)	61.3	(4.2)	60.9	(3.9)
Male Sex (yes)	1863	(49.5)	320	(66.0)
Educational Level				
Primary School	1032	(27.4)	126	(26.0)
Lower Vocational School	802	(21.3)	104	(21.4)
Intermediate Vocational School	1359	(36.1)	162	(33.4)
Higher Vocational School or University	557	(14.8)	91	(18.8)
Unknown	17	(0.4)	2	(0.4)
Lifestyle characteristics				
Energy intake (kcal/day)	1922	(509)	1997	(518)
Dietary fibre intake (g/day)	26.8	(6.7)	27.8	(6.9)
Red and processed meat intake (g/day)	99.2	(41.4)	100.7	(43.2)
Ratio of poly-unsaturated to saturated fat	0.54	(0.27)	0.58	(0.28)
Intake of transfats (g/day)	2.92	(1.15)	2.84	(1.20)
Glycaemic load (g/day)	119.2	(26.8)	123.2	(27.0)
Vegetables and fruits intake (g/day)	365	(151)	351	(150)
Use of alcohol (yes)	2883	(76.5)	376	(77.5)
Intake of alcohol (consumers only, g/day)	13.6	(15.1)	15.2	(15.4)
Cigarette smoking				
Never	1362	(36.2)	124	(25.6)
Former	1390	(36.9)	206	(42.5)
Current	1015	(26.9)	155	(32.0)
Number of cigarettes (users only, n/day)^a^	15	(10–20)	15	(10–20)
Years of cigarette smoking (users only)	38.8	(9.6)	39.9	(8.1)
Non-occupational physical activity (min/day)^a^	58	(34–94)	58	(34–94)
Body mass index (kg/m^2^)	25.0	(3.1)	25.5	(3.1)
Intake of sodium (mg/day)	2315	(617)	2462	(671)
Intake of potassium (mg/day)	3516	(588)	3611	(618)
Intake of magnesium (mg/day)	311	(56)	323	(57)
Medical characteristics				
History of Hypertension and/or History of Antihypertensive Drug Use (yes)	1199	(31.8)	170	(35.1)
History of Diabetes (yes)	123	(3.3)	22	(4.5)
History of Kidney Stones (yes)	326	(8.7)	69	(14.2)
Familial History of Cancer (yes)	1524	(40.5)	208	(42.9)
Familial History of Renal Cancer (yes)	40	(1.1)	6	(1.2)

Continuous variables are displayed as mean (standard deviation) or median (interquartile range). Categorical variables are displayed as number (group percentage)

*RCC* Renal Cell Cancer, *HLI* Healthy Lifestyle Index, *kcal* kilocalories, *kg* kilogram, *m*^2^ squared meter, *min* minutes, *g* gram, *mg* milligram

^a^Continuous variables were not normally distributed

The HLI score was treated as a unitless ratio-scale variable and ranged between 0 and 20, with higher scores reflecting healthier lifestyles. HLI scores were recoded into four categories. Cut-off values were chosen to strive for categories distributed as evenly as possible in the subcohort. As such, the following categories were created: category 1 for HLI scores 0–7, category 2 for HLI scores 8–10, category 3 for HLI scores 11–13 and category 4 for HLI scores 14–20.

### Statistical analyses

Baseline characteristics were described for cases and subcohort members. Continuous variables were described with mean (SD) in case of normal distribution or with median (IQR) in case of non-normal distribution. In addition, categorical variables were described using frequency (%). Moreover, baseline characteristics were described per HLI category within the subcohort. Differences between HLI categories were tested with the Chi-Square test for categorical variables and with the Kruskal–Wallis test for continuous variables.

Hazard ratios and 95%-confidence intervals were estimated using multivariable-adjusted Cox proportional hazard models. Person-time at risk was calculated as the number of years between the start of the NLCS (1986) and year of RCC registration, year of death, year of loss to follow-up or year of end of follow-up, whichever occurred first. In the primary analysis, the association between HLI score category and RCC risk was examined. The analysis was first unstratified and later stratified for sex, alcohol consumption, history of hypertension and history of kidney stones in order to investigate whether the associations between HLI score and RCC risk differ according to strata of these determinants. The stratification according to alcohol consumption was conducted because alcohol consumption is associated with a decreased risk of RCC. Effect modification by these determinants was checked with the Wald χ2-test. Next, measured covariates were included as confounders in the multivariable model if these covariates changed the hazard ratios with more than 10%. Covariates that were considered as potential confounders were sex, diagnosis of hypertension, diagnosis of diabetes, dietary intake of sodium and magnesium. Age at baseline was locked as confounder. For continuous analyses, increments of 1-SD increases in HLI score were examined. Ordinal exposure variables were fitted as continuous variables in trend analyses. To investigate whether the relationship between HLI score and RCC risk is linear, restricted cubic splines were created with three knots (at p5, p50 and p95) to graphically present the dose–response curves without making a priori assumptions about the shapes. Wald tests were performed to evaluate the linearity of these relationships.

Following, in the secondary analysis, the HLI score was recomputed by eliminating each component score one at a time. Subsequently, increments of 1-SD increases in the recomputed HLI scores and RCC risk were checked.

Next, a sensitivity analysis was conducted by excluding the first two years of follow-up and repeating the primary analysis. This analysis aims to check whether hazard ratios did not differ with more than 10% from the primary analysis. If so, then it was concluded that reverse causality may have affected the study results. Furthermore, a sensitivity analysis was conducted excluding cohort participants who were underweight at baseline (body mass index < 18.5 kg/m^2^), as this is often associated with an increased cancer risk. Finally, a sensitivity analysis was conducted adding history of hypertension as a covariate in the multivariable analysis to investigate whether hypertension acts as an intermediary in the association between HLI score and RCC risk.

For all analyses, the proportional hazard assumption was checked. This assumption was checked using the scaled Schoenfeld residuals and log–log transformations of survival curves. The proportional hazard assumption was violated by age. As such, age was added as a time-dependent covariate in the Cox model. The results of the Cox model with age as time-depending covariate were compared to the results of a Cox model in which age-on-study was used as timescale. The comparison of these results is addressed in Additional file 1: Appendix 1.3. Moreover, attributable to the case-cohort study design of the NLCS and the consequent sampling of the subcohort, additional variance into the analysis was introduced. To account for this, the robust Huber-White sandwich estimator was used to calculate standard errors. This correction is similar to Barlow’s variance–covariance estimator [33]. Individuals with missing data were excluded from data analysis.

All statistical tests were performed two-sided. A probability value of 0.05 was used as cut-off value to assess statistical significance. The analyses were carried out using STATA software (Version 16.1 StataCorp., College Station, USA.).

## Results

### Baseline characteristics

The study population consisted of 485 RCC cases and 3,767 subcohort members with complete information (Table 1). Cases were slightly younger than subcohort members and more often men.

In addition, cases slightly consumed more calories and more alcohol than subcohort members. Prevalence of smoking was higher among cases, but median smoking frequency among consumers did not differ. Next, time spent on non-occupational PA was identical to the subcohort and BMI was slightly higher in cases.

Age at baseline was significantly higher in higher/healthier HLI categories within the subcohort (*p* = 0.002) (Table 2). Moreover, the proportion of male sex decreased with higher HLI categories (*p* < 0.001). As expected, exposures associated with an unhealthy lifestyle, e,g, dietary intakes, high alcohol consumption, high cigarette consumption, low amount of PA and high BMI were more observable in lower/unhealthier HLI categories. Sodium intake was lower (*p* = 0.01) in higher HLI categories, while potassium and magnesium intakes were higher (both* p* < 0.001). The report of history of hypertension, prescription of antihypertensive drugs and history of diabetes did not differ between HLI categories (*p* = 0.35, *p* = 0.13, *p* = 0.65, respectively). The proportions of history of kidney stones did not differ significantly between the four categories (*p* = 0.10*).*

**Table 2:** Baseline characteristics per Healthy Lifestyle Index Score Category of the subcohort in the Netherlands Cohort Study on Diet and Cancer (1986–2006). Differences between categories were tested with the Chi-Square test for categorical variables and with the Kruskal–Wallis Test for continuous variables

	HLI categories								
Baseline Characteristics	1 (*n* = 541)		2 (*n* = 1023)		3 (*n* = 1352)		4 (*n* = 851)		*p*-value
Demographic Characteristics									
Age at Baseline (years)	60.8	(4.2)	61.3	(4.2)	61.3	(4.2)	61.5	(4.3)	0.002
Male Sex (yes)	402	(74.3)	597	(58.4)	598	(44.2)	266	(31.3)	< 0.001
Educational Level									
Primary School	155	(28.7)	308	(30.1)	352	(26.0)	217	(25.5)	0.18
Lower Vocational School	109	(20.1)	205	(20.0)	298	(22.0)	190	(22.3)	
Intermediate Vocational School	184	(34.0)	353	(34.5)	493	(36.5)	329	(38.7)	
Higher Vocational School or University	89	(16.5)	150	(14.7)	205	(15.2)	113	(13.3)	
Unknown	4	(0.7)	7	(0.7)	4	(0.3)	2	(0.2)	
Lifestyle characteristics									
Energy intake (kcal/day)	2057	(537)	1958	(521)	1886	(505)	1851	(460)	
Dietary fibre intake (g/day)	23.0	(5.4)	25.3	(5.8)	27.7	(6.7)	29.9	(6.6)	
Red and processed meat intake (g/day)	115.4	(40.5)	105.6	(40.0)	98.3	(39.6)	82.9	(41.1)	
Ratio of poly-unsaturated/saturated fat	0.45	(0.21)	0.50	(0.24)	0.56	(0.27)	0.64	(0.30)	
Intake of transfats (g/day)	3.23	(1.55)	3.05	(1.15)	2.87	(1.05)	2.63	(0.90)	
Glycaemic load	122.4	(28.9)	121.3	(27.3)	117.9	(26.6)	116.6	(24.7)	
Vegetables and fruits intake (g/day)	272	(111)	323	(129)	383	(146)	446	(157)	
Use of alcohol (yes)	515	(95.2)	855	(83.6)	994	(73.5)	519	(61.0)	
Intake of alcohol (consumers only, g/day)	23.4	(19.8)	15.6	(14.9)	10.9	(12.2)	6.0	(7.6)	
Cigarette smoking									
Never	21	(3.9)	191	(18.7)	576	(42.6)	574	(67.5)	
Former	157	(29.0)	431	(42.1)	555	(41.1)	247	(29.0)	
Current	363	(67.1)	401	(39.2)	221	(16.3)	30	(3.5)	
Number of cigarettes (users only, /day)^a^	20	(15–25)	15	(10–20)	10	(7–15)	8	(5–10)	
Years of cigarette smoking (users only)									
Non-occupational physical activity (min/day)^a^	34.3	(19.3–49.3)	47.1	(30.0–75.7)	64.3	(41.4–94.3)	88.6	(60.0–124.3)	
Body mass index (kg/m^2^)	26.4	(3.1)	25.4	(3.1)	24.9	(2.9)	23.5	(2.6)	
Intake of sodium (mg/day)	2364	(629)	2319	(623)	2319	(613)	2273	(605)	0.01
Intake of potassium (mg/day)	3420	(559)	3462	(558)	3544	(599)	3596	(610)	< 0.001
Intake of magnesium (mg/day)	293	(51)	303	(50)	315	(57)	326	(58)	< 0.001
Medical characteristics									
History of Hypertension and/or History of Antihypertensive Drug Use (yes)	159	(29.4)	321	(31.4)	447	(33.1)	272	(32.0)	0.47
History of Diabetes (yes)	17	(3.1)	29	(2.8)	44	(3.3)	33	(3.9)	0.65
History of Kidney Stones (yes)	47	(8.7)	107	(10.5)	107	(7.9)	65	(7.6)	0.10
Familial History of Cancer (yes)	236	(43.6)	403	(39.4)	530	(39.2)	355	(41.7)	0.36
Familial History of Renal Cancer (yes)	3	(0.6)	14	(1.4)	18	(1.3)	5	(0.6)	0.17

Variables incorporated in the Healthy Lifestyle Index were not tested on significant differences. Continuous variables were displayed as mean (standard deviation) or median (interquartile range). Categorical variables were displayed as number (group percentage)

*HLI* Healthy Lifestyle Index, *kcal* kilocalories, *kg* kilogram, m^2^ squared meter, *mg* milligram

^a^Continuous variables were not normally distributed

### Healthy lifestyle index category

Sex and hypertension were no significant effect modifiers (*p* = *0.19*, *p* = *0.62*, respectively) of the association between HLI score and RCC risk. However, sex fulfilled the conditions to be included as a confounder. Other potential confounders did not change the hazard ratios with > 10% and were therefore not included in the multivariable models. As such, all analyses were adjusted for age at baseline (years) and sex (man/woman).

Results of the unstratified primary analysis shows that, based on the *p* for trend, HLI category was inversely associated with RCC risk (*p* for trend = 0.045) (Table 3). Compared with participants in the unhealthiest HLI category, participants in the healthiest category had a 21% lower RCC risk (HR = 0.79, 95% C.I. = 0.56–1.10). Moreover, a 1-SD (approximately 3 units) increase in HLI score was inversely associated with RCC risk (HR = 0.92, 95% C.I. = 0.83–1.01). The p-test for non-linearity of the total HLI-score and RCC risk was 0.22.

**Table 3:** Renal Cell Cancer risk according to indicators of Healthy Lifestyle Index Category in the Netherlands Cohort Study on Diet and Cancer (1986–2006). The analysis was additionally stratified on sex. HLI Category 1 was considered to be the reference

Healthy Lifestyle Index Score	Median HLI Score in subcohort (men/women)	Cases (n)	Subcohort (n/ person years)	HR^a^	95% CI
Overall					
1 “Unhealthiest”	(6/7)	84	541/8443	1	ref
2 “Moderately Unhealthy”	(9/9)	158	1023/17017	1.04	(0.78–1.39)
3 “Moderately Healthy”	(12/12)	157	1352/23333	0.83	(0.62–1.11)
4 “Healthiest”	(15/15)	86	851/14944	0.79	(0.56–1.10)
*P* for trend					0.045
Increment per 1-SD unit score		485	3767/63737	0.92	(0.83–1.01)
Men^b^					
1 “Unhealthiest ”	(6)	67	402/6117	1	ref
2 “Moderately Unhealthy”	(9)	112	597/9590	1.07	0.76–1.49
3 “Moderately Healthy”	(12)	105	598/9767	0.98	0.70–1.36
4 “Healthiest”	(15)	36	266/4271	0.76	0.49–1.18
*P* for trend					0.23
Increment per 1-SD unit score		320	1863/29745	0.94	0.84–1.06
Women^b^					
1 “Unhealthiest”	(7)	17	139/2326	1	ref
2 “Moderately Unhealthy”	(9)	46	426/7427	0.85	0.47–1.55
3 “Moderately Healthy”	(12)	52	754/13566	0.53	0.29–0.94
4 “Healthiest”	(15)	50	585/10673	0.64	0.36–1.15
*P* for trend					0.07
Increment per 1-SD unit score		165	1904/33992	0.86	0.73–1.03

*HR* Hazard Ratio, *CI* confidence interval, *SD* standard deviation, *ref* reference

^a^Adjusted for age at baseline (years) and sex (male/female)

After stratification on sex (*p* for interaction, 0.19), the association between HLI score and RCC risk was slightly stronger in women than in men (Table 3 and Fig. 3). A 1-SD increase in HLI score was associated with a HR of 0.94 (95%CI 0.84–1.06) in men and 0.86 (95%CI 0.73–1.03) in women. The association between HLI score and RCC risk (*p* for interaction, 0.79) was slightly stronger in participants who did not report to consume alcohol (HR 0.88; 95%CI 0.71–1.09) than in participants who reported to consume alcohol (HR 0.91; 95%CI 0.81–1.01). After stratification on a history hypertension (*p* for interaction, 0.62), no large differences in associations with the unstratified analysis were observable. The association of HLI score and RCC risk, was different according to history of kidney stones (*p* for interaction, 0.04). The association between HLI score and RCC risk was statistically significant inverse (HR 0.69; 95%CI 0.53–0.92) in participants who reported a history of kidney stones.

As sensitivity analysis, we tested whether hypertension was an intermediate of the association between HLI and RCC risk. No differences in the multivariable-adjusted models were observed after adding hypertension in the model (data not shown). Excluding cohort participants with a body mass index < 18.5 kg/m^2^ (underweight) did not change the observed associations as well (data not shown).

### Recomputed healthy lifestyle index score

**Fig. 2:**
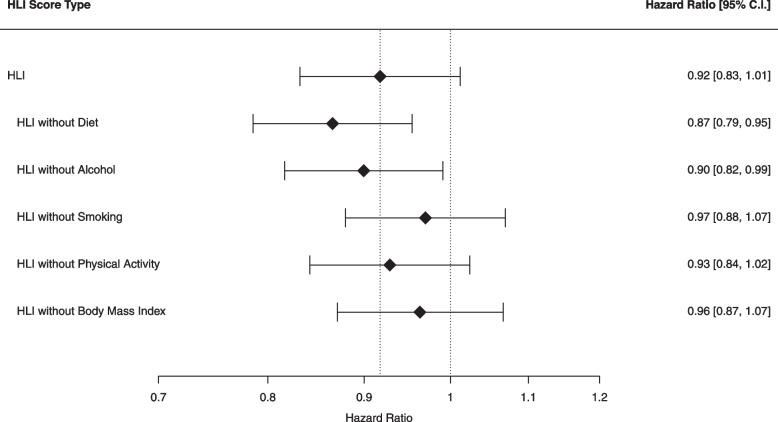
Association between the Healthy Lifestyle Index score and Renal Cell Cancer Risk in the Netherlands Cohort Study (1986–2006). Footnote: Hazard Ratios were calculated per 1-SD increment. The standard deviation was calculated for every HLI score type. The first HLI score type was calculated without subtracting a component score. The other five HLI score types were recalculated by subtracting in turn each component score. One standard deviation corresponded to 2.7–3.2 units of HLI score, depending on the HLI score type. The model was unstratified for hypertension status and adjusted for age at baseline (years) and sex (male/female). HLI = Healthy Lifestyle Index, C.I. = confidence interval

**Fig. 3:**
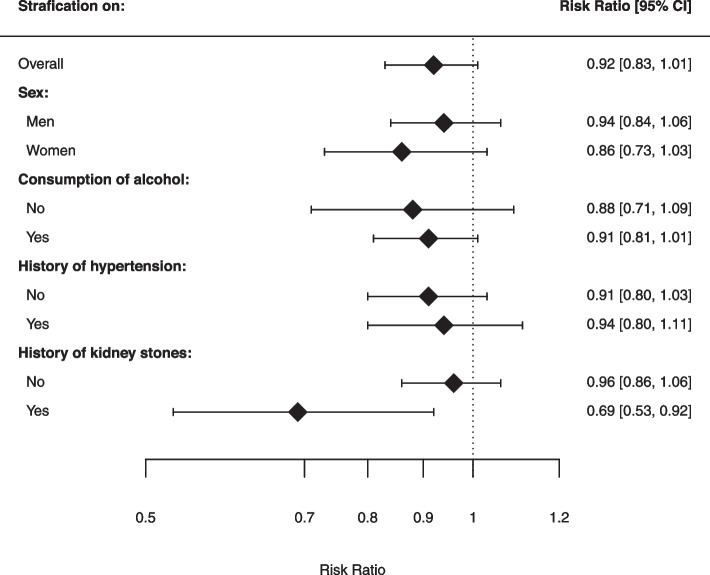
Associations between the Healthy Lifestyle Index score with Renal Cell Cancer Risk, stratified on sex, alcohol consumption, history of hypertension and history of kidney stones. Footnote: Hazard Ratios were calculated per 1-SD increment. The standard deviation was calculated for every HLI score type. One standard deviation corresponded to 2.6–3.3 units of HLI score, depending on the HLI score type. The model was adjusted for age at baseline (years) and sex (male/female) if applicable. HLI = Healthy Lifestyle Index, C.I. = confidence interval

In the secondary analysis, each HLI component was in turn excluded from the HLI and linear trends across 1-SD unit increases in recomputed HLI scores were checked and compared with linear trends across 1-SD unit increases in the original HLI score. Results are shown in Fig. 2. After exclusion of the diet component, the association between HLI score and RCC risk for every 1-SD increase in HLI score turned stronger in the analysis including all participants (HR = 0.87, 95% C.I. = 0.79–0.95). The same pattern was observable after exclusion of the alcohol component (HR = 0.90, 95% C.I. = 0.82–0.99). In contrast, exclusion of the smoking component and the BMI component led to a weaker association between HLI score and RCC risk (HR = 0.97, 95% C.I. = 0.88–1.07; HR = 0.96, 95% C.I. = 0.87–1.07, respectively). Similarly, after exclusion of the non-occupational PA component, the new association between HLI score and RCC risk changed into a slightly weaker association than the original association (HR = 0.93, 95% C.I. = 0.84–1.02). After stratification on hypertension status of the secondary analysis, no large differences with the observed trends in the unstratified analysis were observable. More detailed information on risk estimates after stratification can be found in Fig. 3 and in Additional file 1: Appendix 1.4.

### Exclusion of first two years of follow-up

Exclusion of the first two years of follow-up and repeating the primary analysis did not change the results with more than 10% (data not shown). As such, reverse causation and/or preclinical disease was not likely to have influenced the study results.

## Discussion

This prospective cohort study, that uses a case cohort design, showed a weak inverse association between HLI score and RCC risk. Based on the *p* for trend, there was a statistically significant association between HLI score and RCC risk, while the 1-SD increment in HLI score did not show a statistically significant inverse association between HLI score and RCC risk. Nevertheless, both observations demonstrated that a healthy lifestyle, reflected by a high HLI score, was associated with a lower RCC risk.

The associations between HLI score and RCC risk were not statistically significant different after stratification on sex, alcohol consumption or a history of hypertension. The association between HLI score and RCC risk was statistically significant inverse in participants who reported a history of kidney stones. A change in lifestyle after diagnosis of kidney stones is not likely, although it is possible that these participants have been advised to drink more water to prevent kidney stones. Fluid intake is not associated with RCC risk in the NLCS [34], and fluid intake of participants with a history of kidney stones was only slightly higher than participants without a history of kidney stones (1428 ml/day versus 1399 ml/day). After stratification on hypertension, similar changes in associations to the unstratified analysis were observable. This does not indicate that hypertension acted as intermediate of the association. Up to present, this is the one of the few studies to investigate the joint influence of various lifestyle factors on RCC risk.

Although to our knowledge, only one study studied the association between a Healthy Lifestyle Index and RCC risk [23]. In the prospective Norwegian Women and Cancer (cohort), a HLI score was constructed using the same components. A 1-point increase in HLI score (range 0–20) was associated with a reduced kidney cancer risk (HR 0.94; 95%CI 0.91–0.97). This risk is stronger than the overall risk we observed, although in the stratified analysis according to sex, the HR in the NLCS was 0.94 (95%CI 0.73–1.03) per 1SD increment (~ 3 points), which is comparable to the result in the NOWAC study. The NOWAC study did also observe that BMI is an important contributor to the inverse association, although in contrast to our study, smoking was not a strong contributor to the association between HLI score and kidney cancer risk in the NOWAC study [23].

Various studies have investigated the association of various dietary components, such as red meat, fruits, and vegetables, with RCC risk. Previous studies and meta-analyses reported null associations or contradictory results regarding these dietary components [5, 13, 15, 35, 36]. An earlier publication of a study using NLCS data and a shorter follow-up period showed a null association between total fruit and vegetable intake and RCC risk [37]. Most of the dietary components captured in the HLI were not studied within the NLCS. Consequently, the finding of the current study, in which the relationship between HLI and RCC risk was stronger when diet was omitted from the HLI, is not fully understood. Nevertheless, this finding suggests that a healthy diet, as operationalized in the HLI within this study, is not associated with a lower RCC risk.

Furthermore, several meta-analyses showed a reduced RCC risk of 20–30% among drinkers at the highest level of alcohol intake compared to non-drinkers [16–18]. These observations may explain why the current study showed that the association between HLI and RCC risk was stronger after excluding alcohol consumption from the HLI. A meta-analysis from 2016 showed that current smokers and former smokers have a small increased RCC risk (pooled RR:1.31; 95%CI 1.22–1.40), and this risk positively correlates with smoking frequency [8, 9]. As such, these findings are in agreement with the observation of the current study, in which a shift towards a weaker association between HLI and RCC risk was found after exclusion of smoking from the HLI.

A meta-analysis from 2013, showed a small RCC risk reduction of 13% when comparing participants with a high PA level versus a low PA level [12]. In a large pooled analysis, an inverse association was observed between leisure time physical activity and kidney cancer risk was observed (HR 0.77; 95% CI 0.70–0.85) [14]. Similarly, a study conducted within the NLCS demonstrated that non-occupational PA was inversely, although not statistically significantly, associated with RCC risk in men but not in women [38]. These findings may explain the small shift towards a weaker association between HLI and RCC risk after exclusion of non-occupational PA. It should be noted that this shift is more apparent in participants with hypertension.

Finally, two studies conducted within the NLCS demonstrated that every unit increase in BMI at baseline was positively associated with RCC risk [38, 39]. Moreover, a meta-analysis from 2014 showed that overweight and obese persons had a 28% and 77% higher RCC risk compared to people with a BMI below 25 kg/m^2^ [11]. Considering these studies, their results may reinforce the notion that the weaker association between HLI and RCC risk after exclusion of BMI may be attributable to the positive association between BMI and RCC risk.

The results of the current study and the Norwegian study [23] illustrate that maintaining a healthy lifestyle is associated with lower risk of certain cancers. As such, the HLI might aid as a framework to lower the risk of cancer by encouraging healthy behaviours such as adopting a healthy diet and participating in physical activities while also discouraging unhealthy behaviours such as smoking, consuming alcohol and being overweight/obese. Furthermore, the current study implied an inverse association between alcohol consumption and RCC risk, which may question why alcohol consumption is part of the HLI. However, alcohol consumption is a risk factor for other cancers and it is therefore advisable to keep the alcohol consumption component in the HLI [19–21]. The components of the Healthy Lifestyle Index were chosen because it was implicated that they were associated with a lower risk of cancer and other diseases like cardiovascular disease and diabetes mellitus [22, 24, 25, 40]. Another note that should be made is that HLI scores are only relative within a population and cannot be compared to HLI scores within other populations. This is especially true for diet and PA component scores, as these scores were based on quintiles within the subcohort in our study. Moreover, the data on the HLI components can be measured in various ways and their validity is highly dependent on the quality of the questionnaire. To illustrate, PA was based on non-occupational PA, whereas this may also be based on occupational PA. Consequently, the construction of the HLI has to be adapted to the study population and its exposure(s).

Using an HLI score has limitations that have to be considered. All components have an equal weight, but this may not reflect the strength of the individual associations. Moreover, the score assumes linearity in the units, and this may not reflect the proportional changes in behaviour. Finally, by combining the components into one score, this may cause loss of information that is reflected by the individual components of the score.

The current study had several strengths and weaknesses. To start with the strengths, the study results were not likely to be influenced by information bias through differential recall bias. This can be attributed to the prospective study design. Moreover, selection bias via loss to follow-up was negligible because of the high completeness of cancer follow-up and vital status. The potential for information bias was further reduced by excluding participants with cancer at baseline. The rationale for this was the assumption that participants may change their dietary habits after a diagnosis of cancer, which a.o. was shown to be true with regard to a vegetarian lifestyle [41]. Next, the extensive baseline questionnaire guarded against confounding as data on a vast number of variables was requested. Finally, the length of follow-up resulted in a large number of cases.

Despite the various methodological advantages of this study, there was one potential source of information bias. Data on exposure was collected via self-administered questionnaires. This methodological consideration had two shortcomings. First, exposure was self-reported, which may have resulted in a difference between reported exposure and true exposure. Secondly, measurement of exposure was conducted only at baseline. No information was available on potential changes in exposure over the period of follow-up. Nevertheless, a study on reliability of the SFFQ within the NLCS showed that the dietary habits of the participants remained fairly stable in the period of five years after baseline [30]. However, it is unknown whether habits regarding smoking, PA, BMI and medical conditions have changed. Similarly, it is unknown whether dietary habits and alcohol consumption have changed after the first five years. Moreover, lifestyle changes may have occurred in participants diagnosed with medical conditions (such as hypertension) after baseline. Even so, it is thought that this information bias was non-differential due to the prospective nature of the NLCS. Finally, because of errors and inconsistencies in the food frequency questionnaire and missing data in components of the HLI score, 20% of the cases and 21% of the subcohort members could not be included in the analysis. This has decreased the statistical power of the analysis, and if exclusions are not completely at random, may have caused bias. However, because the proportion of cases and subcohort members is quite similar, bias is less likely.

## Conclusions

This study suggests a weak inverse relationship between adherence to a healthy lifestyle, reflected by a high HLI score, and RCC risk in Dutch adults. Adopting a healthy lifestyle might be an effective strategy to prevent renal cell cancer.

## Supplementary Information

**Additional file 1: Appendix.** 

## Data Availability

The datasets generated and/or analysed during the current study are not publicly available because the informed consent does not allow for that. However, anonymous data that are minimally required to replicate the outcomes of the study will be made available upon reasonable request and approval by the institutional review boards. Requests can be addressed to Piet A. van den Brandt, e-mail: pa.vandenbrandt@maastrichtuniversity.nl.

## Abbreviations

HLI: Healthy Lifestyle Index
RCC: Renal Cell Cancer
NLCS: Netherlands Cohort Study
PALGA: Netherlands Pathology Registry
NCR: Netherlands Cancer Registry
SFFQ: Semi-Quantitative Food Frequency Questionnaire
PA: Physical Activity
BMI: Body Mass Index
HR: Hazard Ratio
C.I.: Confidence Interval
(1-)SD: (1) Standard Deviation
IQR: Interquartile Range
