# Supplementary material for: The association of healthy lifestyle index score and the risk of renal cell cancer in the Netherlands cohort study
Source: BMC Cancer. 2023 Feb 16;23:156. doi: 10.1186/s12885-023-10627-6 (PMC9933336; doi:10.1186/s12885-023-10627-6)
Supplement: Supplementary file 1 — Additional file 1: Appendix. [file 12885_2023_10627_MOESM1_ESM.docx]

The Association of Healthy Lifestyle Index Score and the Risk of Renal Cell Cancer among Men and Women in the Netherlands Cohort Study

Romain Meer ^(1)^, Jeroen van de Pol ^(1)^, Piet A. van den Brandt ^(1,2)^, Leo J. Schouten ^(1)^

**Additional file 1: Appendix**

- 1. *The Netherlands Cohort Study on Diet and Cancer (NLCS)*

The NLCS consisted of 120,852 participants (48.2% men) aged 55-69 years at baseline. The study population was recruited via sex-stratified random sampling using 204 Dutch municipal population registries. An invitation was sent to 340,439 individuals and the response rate was therefore 35.5%. Individuals aged 55-69 years in 1986 were invited to participate in the NLCS because it has been hypothesized that diet mainly plays a role in late stages of tumorigenesis rather than in early stages of tumorigenesis. (39) Moreover, dietary habits tended to be unstable in younger individuals and also in the age group above 70 years. (22)

Dietary habits were measured with the SFFQ. The validity and reliability of the SFFQ have been tested within the NLCS in a period of respectively two and five years after baseline. (25,26) Results of the study on reliability showed that the test-retest correlation ranged between 0.42 and 0.90 for nutrient intake after five years, indicating that dietary habits within the NLCS remained fairly stable over a time period of five years. (26) Results of the study on validation showed that the correlation regarding nutrient intake obtained from the SFFQ and from a dietary record ranged between 0.40 and 0.86, with correlations for most nutrients being within the range of 0.60 and 0.80. (25) This showed that the SFFQ provides valid results regarding nutrient intake within the NLCS.

*1.2 Healthy Lifestyle Index Component Scores*

Detailed description of computing diet scores is described elsewhere. (18) In short, the diet score was based on the energy-adjusted intake of six dietary components: fibre, red and processed meat, the ratio of poly-unsaturated to saturated fat, trans-fats, glycaemic load, and fruits and vegetables. The energy-adjusted intake for each dietary component was divided into deciles ranging from 0 (lowest dietary component intake) to 9 (highest dietary component intake) based on data from the subcohort. For meat, trans-fats and glycaemic load, the inverse decile scores were calculated. Then, the sum-scores were calculated over all dietary components (ranging from 0 to 54) and these sum-scores were divided into sex-specific quintiles. Following this, both subcohort members and cases were assigned a diet score between 0 (unhealthiest diet) and 4 (healthiest diet) according to their sex-specific quintile (see Table A1). The alcohol consumption score was based on the daily intake of alcohol in grams and the cut-off values can also be found in Table A1.

In addition to the daily food intake and alcohol consumption, the questionnaire measured amongst others non-occupational physical activity, smoking habits and anthropometric measurements needed for the calculation of the body mass index. Non-occupational physical activity was quantified in minutes per day and was based on frequency and duration of cycling, walking, gardening, and sports. Then, the index score was computed according to sex-specific quintiles of physical activity in minutes per day. The smoking score was based on smoking status (current, ex-smoker, never smoked), years of abstinence and daily consumption of cigarettes. Body mass index was calculated by dividing the self-reported weight at baseline in kilograms (kg) by the self-reported height at baseline in meters squared (m^2^) and the index scores were computed based on the cut-off values as was used in the McKenzie study. ^(18)^

The approach to determine the cut-off values for the components of diet, non-occupational physical activity, smoking and body mass index within this study were identical to the approach in the McKenzie study. (18) The cut-off values chosen for the alcohol consumption component differed from the cut-off values as picked by McKenzie et al, because using the prespecified cut-off values would have yielded a skewed distribution with floor effects. As such, this study maintained different cut-off values for alcohol consumption. All cut-off values as used in this study are shown in Table A1.

After determining every component score for the five lifestyle factors, the HLI score was calculated by summing all five individual component scores. Subsequently, the HLI score category was determined with the cut-off values as mentioned in the methods section.

*1.3 Time-depending Covariates versus Age-on-Study*

In the methods section, it was mentioned that either age-on-study was used, or time-depending covariates were added to the model in case of violation of the proportional hazard assumption. In this study, both solutions were compared with each other. During all analyses, the proportional hazard assumption for age at baseline was violated. As such, age was either entered in every model as a time-depending covariate or the age-on-study approach was used. For the second approach, the Cox proportional hazard models were no longer adjusted for age at baseline. However, these models were additionally adjusted for birth cohort, depending on year of birth (1916-1920, 1921-1925 or 1926-1931), since cohort effect was plausible. Comparing the results of both approaches with each other did not reveal any large differences (data not shown). The differences in results of both approaches were maximally 5% with most differences between 1-3%. Moreover, the differences were multidirectional (equally towards the null as away from the null). Because of computational simplicity, the approach of adding time-depending covariates in the model was preferred over the age-on-study approach.

*1.4 Recomputed Healthy Lifestyle Index Score: stratified on Hypertension Status*

The secondary analysis with the recomputed HLI scores was also stratified on hypertension status (see Figure 3). After exclusion of the diet component score from the HLI, the association between HLI and RCC risk became stronger for both participants without hypertension (from HR=0.91, 95% C.I.=0.80-1.03 to HR=0.85, 95% C.I.=0.75-0.96) as well as participants with hypertension (from HR=0.94, 95% C.I.=0.80-1.11 to HR=0.91, 95% C.I.=0.78-1.07). It should be noted that the magnitude of change was larger in participants without hypertension. Concerning exclusion of the alcohol component score, the association between HLI and RCC risk remained unchanged for participants without hypertension (HR=0.91, 95% C.I.=0.81-1.03), whereas the association became stronger in participants with hypertension (HR=0.89, 95% C.I.=0.76-1.05). In sharp contrast with the exclusion of diet and alcohol from the HLI, omitting the smoking component score led to a weaker association between HLI and RCC risk for both participants without hypertension (HR=0.98, 95% C.I.=0.87-1.11) and with hypertension (HR=0.97, 95% C.I.=0.82-1.14). Next, omitting the physical activity component score did not change the association between HLI and RCC risk in non-hypertensive participants (HR=0.91, 95% C.I.=0.80-1.03), but weakened the same association in hypertensive participants (HR=0.97, 95% C.I.=0.83-1.14). Finally, exclusion of the body mass index component score from the HLI weakened the association between HLI and RCC risk in both non-hypertensive participants (HR=0.93, 95% C.I.=0.82-1.06) and hypertensive participants (HR=1.01, 95% C.I.=0.85-1.19), although the degree of change is larger in the latter subgroup.

**Table A1:** Cut-off values for each Healthy Lifestyle Index component. Netherlands Cohort Study on Diet and Cancer.

| **HLI Component Score**  (*unitless)* | **Diet Quintile Score**  (*unitless)* | **Alcohol Consumption**  *(gram / day)* | **Smoking Status**  *(unitless)* | **Non-occupational Physical Activity**  *(minutes / day)* | **Body Mass Index**  *(kg / m^2^)* |
| --- | --- | --- | --- | --- | --- |
| 0 | M: 5 - 19  W: 4 - 19 | ≥ 30.0 | Current smoker,  > 15 cigarettes per day | M: 0 – 32.9  F: 0 – 26.9 | ≥ 30.0 |
| 1 | M: 20 - 25  W: 20 - 25 | 15.0 – 29.9 | Current smoker,  ≤ 15 cigarettes per day | M: 33.0 – 50.9  F: 27.0 – 43.9 | 26.0 – 29.9 |
| 2 | M: 26 - 29  W: 26 - 29 | 5.0 – 14.9 | Ex-smoker,  quit ≤ 10 years | M: 51.0 – 78.9  F: 44.0 – 65.9 | 24.0 – 25.9 |
| 3 | M: 30 - 34  W: 30 - 34 | 0.1 – 4.9 | Ex-smoker,  quit > 10 years | M: 79.0 – 115.9  F: 66.0 – 92.9 | 22.0 – 23.9 |
| 4 | M: 35 - 51  W: 35 - 54 | 0 | Never smoked | M: > 116.0  F: > 93.0 | < 22.0 |

HLI = Healthy Lifestyle Index, M = men, W= women, kg = kilogram, m^2^ = squared meter
